# Time-series representation learning via Time-Frequency Fusion Contrasting

**DOI:** 10.3389/frai.2024.1414352

**Published:** 2024-06-12

**Authors:** Wenbo Zhao, Ling Fan

**Affiliations:** ^1^International School, Beijing University of Posts and Telecommunications, Beijing, China; ^2^School of Electronic Engineering, Beijing University of Posts and Telecommunications, Beijing, China

**Keywords:** representation learning, time-domain augmentation, frequency-domain augmentation, self-supervised learning, contrastive learning

## Abstract

Time series is a typical data type in numerous domains; however, labeling large amounts of time series data can be costly and time-consuming. Learning effective representation from unlabeled time series data is a challenging task. Contrastive learning stands out as a promising method to acquire representations of unlabeled time series data. Therefore, we propose a self-supervised time-series representation learning framework via Time-Frequency Fusion Contrasting (TF-FC) to learn time-series representation from unlabeled data. Specifically, TF-FC combines time-domain augmentation with frequency-domain augmentation to generate the diverse samples. For time-domain augmentation, the raw time series data pass through the time-domain augmentation bank (such as jitter, scaling, permutation, and masking) and get time-domain augmentation data. For frequency-domain augmentation, first, the raw time series undergoes conversion into frequency domain data following Fast Fourier Transform (FFT) analysis. Then, the frequency data passes through the frequency-domain augmentation bank (such as low pass filter, remove frequency, add frequency, and phase shift) and gets frequency-domain augmentation data. The fusion method of time-domain augmentation data and frequency-domain augmentation data is kernel PCA, which is useful for extracting nonlinear features in high-dimensional spaces. By capturing both the time and frequency domains of the time series, the proposed approach is able to extract more informative features from the data, enhancing the model's capacity to distinguish between different time series. To verify the effectiveness of the TF-FC method, we conducted experiments on four time series domain datasets (i.e., SleepEEG, HAR, Gesture, and Epilepsy). Experimental results show that TF-FC significantly improves in recognition accuracy compared with other SOTA methods.

## 1 Introduction

Time series plays fundamental roles in many areas, such as financial markets, clinical diagnosis, and climate science (Harutyunyan et al., 2019; Mahmud et al., 2020; Ravuri et al., 2021). Time series mining is a pivotal tool for comprehending the objective world and natural phenomena but also for informing crucial scientific decisions. Consequently, the study of time series has garnered substantial interest from both industrial and academic research communities alike.

In the last 10 years, deep learning models have demonstrated superior performance when contrasted with traditional machine learning methods in handling time series data (Dempster et al., 2020; Sun et al., 2021). Deep neural networks demonstrate remarkable efficacy, especially when trained with large amounts of labeled data. However, time series patterns typically lack human recognizable characteristics and require specialists for labeling. Hence, labeling time-series data is more arduous than labeling images, resulting in a lack of labeled time-series data in real-world applications (Ching et al., 2018). Consequently, acquiring large amounts of labeled time series data presents a significant challenge, as it is a costly and time-consuming process. Moreover, the human-driven label annotation process is vulnerable to inherent biases, potentially resulting in ambiguous annotations. To address the label challenge, numerous algorithms have emerged, including semi-supervised learning, weakly-supervised learning, and transfer learning techniques. These approaches aim to mitigate the dependency on labeled data, offering promising avenues for alleviating the labeling burden (Qian et al., 2019, 2021; Buffelli and Vandin, 2021). Using these methodologies, researchers can improve the precision and adaptability of deep learning models while mitigating the costs and time constraints in manual labeling procedures.

Self-supervised learning has garnered more attention for extracting effective representations from unlabeled data for downstream tasks involving designing a pretext task and automatically generating intrinsic ground-truth labels for that task. Comparatively, self-supervised pre-trained models, when confronted with limited labeled data, achieve performance on par with supervised models trained on full labeled data (Chen et al., 2020). In response to the distinctive characteristics of time series data, several pretext tasks have emerged, such as masked reconstruction (Haresamudram et al., 2020) and data transformation predictions (Saeed et al., 2019). Through training models with pretext tasks, the acquisition of versatile latent representations can improve the performance of subsequent downstream tasks. An extensively used self-supervised technique is contrastive learning, which involves applying metric learning to instance-level classification tasks (Tian et al., 2021; Park et al., 2022). In this method, metric learning is utilized for instance-level classification tasks, aiming to pre-train a model by contrasting various views of a particular data instance with those of other instances. Demonstrating effectiveness, this strategy enables deep neural networks to acquire robust representations from extensive, unlabeled datasets, avoiding the necessity for labor-intensive manual labels. Furthermore, contrastive learning finds extensive application in computer vision tasks like image classification (Park et al., 2022), anomaly detection (Tian et al., 2021), and graph mining (Zhu et al., 2021), along with applications in natural language processing (Guo et al., 2022). Notably, it attains state-of-the-art performance by employing instance discrimination as its pretext task, surpassing even supervised learning approaches in downstream classification tasks in accuracy. In the realm of time series analysis, contrastive learning has gained comparatively less attention compared to other domains (Liu et al., 2021; Zhang et al., 2022). This is partially attributed to the challenge of identifying appropriate augmentation methods that capture crucial invariance properties within time series data. Most prevailing methods center solely on modeling the time domain, neglecting the frequency domain. This oversight may lead to the omission of crucial information, potentially reducing the efficacy of learned representations in downstream tasks.

In this paper, we introduce TF-FC, a new self-supervised contrastive framework that merges time-domain augmentation techniques like jittering, scaling, permutation, and masking with frequency-domain augmentations such as phase shifting, low-pass filtering, etc., designed for time series classification. The objective of time-domain augmentation is aimed at capturing a variety of temporal characteristics of the time series data by applying methods that include but are not limited to jittering, scaling, permutation, etc., while frequency-domain augmentation is to capture data features by focusing on spectral properties by applying methods that including but not limited phase shift, low pass filter, etc. This approach is particularly advantageous for time series classification tasks, as different classes often exhibit distinct frequency components. Our approach successfully captures temporal and spectral characteristics within time series data, mitigating the need for extensive labeled data. The contribution of our paper is 3-fold:

- We propose a novel self-supervised contrastive framework named TF-FC, which combines time-domain augmentation methods with frequency-domain augmentation methods for time series classification tasks.
- Specifically, TF-FC effectively captures both the temporal and spectral attributes of time series data, enhancing the model's ability to accurately distinguish between different classes. The integration of time-domain and frequency-domain methodologies in our approach represents an innovative and pioneering contribution to the field of time series classification tasks.
- Extensive experimentation on four benchmark datasets showcases the effectiveness of our proposed framework, achieving state-of-the-art performance. These experimental results show the efficacy of our approach and emphasize the potential of self-supervised learning and time-frequency fusion for enhancing time series classification tasks.

The remainder of this paper is structured as follows: In Section 2, we delve into the related work, followed by an extensive description of the TF-FC method framework in Section 3. Section 4 outlines the experiments conducted on the benchmark dataset. Finally, Section 5 is the conclusion and offers insights into future works.

## 2 Related work

### 2.1 Self-supervised learning

Recent advances in self-supervised learning began using pretext tasks on images to create useful representations. These pretext tasks include solving jigsaw puzzles (Noroozi and Favaro, 2016), image colorization (Zhang et al., 2016), and predicting image rotation (Gidaris et al., 2018). While these pretext tasks yielded promising results, their reliance on pretext tasks could potentially constrain the generality of the acquired representations. A generative model can also execute the pretext task. This self-supervised model based on generative modeling can be trained to reconstruct the initial input, thus acquiring valuable representations. For instance, the autoencoder (Vincent et al., 2008) is trained to reconstruct input images. The context encoder (Pathak et al., 2016) is designed to restore missing portions of the input image when a mask is applied. Another commonly used framework in training self-supervised models is contrastive learning. For example, MoCo (He et al., 2020) implemented a momentum encoder to acquire representations from negative pairs retrieved from a memory bank. SimCLR (Chen et al., 2020) replaced the momentum encoder by employing an expanded batch of negative pairs. BYOL (Grill et al., 2020) attained representations by bootstrapping from existing representations without the need for negative samples. SimSiam (Chen and He, 2021) advocated for disregarding negative samples and instead relied solely on a Siamese network and stop-gradient operation to achieve state-of-the-art performance.

Self-supervised representation learning for time series has been becoming more popular recently. Some approaches employed pretext tasks for time-series data. For example, Saeed et al. (2019) devised a binary classification pretext task for human activity recognition. They achieved this by applying multiple transformations to the data and training the model to distinguish between the original and the transformed versions. Sarkar and Etemad (2020) introduced SSL-ECG, a method where ECG representations are acquired through six applied transformations on the dataset serving as pretext tasks. Pseudo-labels are then assigned based on the type of transformation. Saeed et al. (2021) adopted a similar methodology, designing eight auxiliary tasks to learn representations from multi-sensor human activity data. Aggarwal et al. (2019) acquired subject-invariant representations by modeling local and global activity patterns.

### 2.2 Contrastive learning for time series

Contrastive learning, a widely adopted self-supervised learning approach, seeks to train an encoder that maps inputs onto an embedding space. The objective is to minimize the distance between positive sample pairs (comprising the original augmentation and an alternative view of the same input) while maximizing the distance between negative sample pairs (consisting of the initial augmentation and an alternative augmentation of a different input sample). Exploration of contrastive learning in time series remains relatively less compared with other domains, such as image and NLP, etc., primarily because of the difficulty in identifying augmentation methods that effectively capture crucial invariance properties within time series data. For time invariance, Kiyasseh et al. (2021) leverage unlabeled physiological data to derive representations of instances across spatial, temporal, and patient dimensions. Their approach encourages the similarity of these representations while defining adjacent time segments as positive pairs. Tonekaboni et al. (2021) operates assuming that overlapping temporal neighborhoods exhibit comparable representations. These methodologies capitalize on temporal invariance to establish positive pairs, subsequently employed in computing the contrastive loss. For transformation invariance, Tang et al. (2020) assessed eight data augmentation techniques specifically for time series data, replacing traditional image augmentation operators within the SimCLR model, Liu et al. (2021) implemented time-domain and frequency-domain augmentation techniques within the SimCLR framework. Wang et al. (2022) examined the effectiveness of sensor sampling frequencies and introduced a data augmentation method centered on re-sampling within their investigation. Recently, there are multi-invariance methods proposed, Yue et al. (2022) focused on the hierarchy within identical time series. This approach aimed to differentiate multi-scale contextual information at both the timestamp and instance levels.

Several methodologies incorporated frequency domain characteristics to enrich the learning of time-series representations. For instance, the Bilinear Temporal-Spectral Fusion (BTSF) method implemented an iterative bilinear fusion technique, combining feature embeddings from both time series representations. Likewise, both TF-C (Zhang et al., 2022) and STFNets (Liu et al., 2021) acquired representations by encouraging proximity between time domain and frequency domain representations of identical samples while pushing them apart from representations of other signals. Differently, we use the kernel PCA to fuse time-domain augmentation data and frequency-domain augmentation data in data layer. We also use the fused augmented data and original data as positive samples.

## 3 Methodology

### 3.1 Formulation

Our objective aims at solving the time series classification issue. Specifically, when presented with a dataset $D=\left(Xi,y_{i}\right)\left(i\in 1,2,\cdots \;,N\right)$, where $X_{i}\in {\mathbb{R}}^{T\times S}$ represents a multivariate time series with a length of *T* and consisting of *S* sensor channels, and *y*_*i*_ denotes the associated class label, our aim is to train a mapping function *f*:ℝ^*T*×*S*^→*Y*. This function should accurately predict the class label for new, unseen time series. In simpler terms, when presented with a test time series X^*^∈ℝ^*T*×*S*^, the model's output, ŷ = *f*(X^*^)∈*Y*, should match the true class label *y*.

The paper employs the self-supervised learning method to pre-train an encoder with unlabeled data. While class labels are solely utilized for fine-tuning the model, the initial pre-training stage operates self-supervised without the need for class labels.

In this scenario, the encoder, trained in the pretext task, can be regarded as a function *f*:ℝ^*T*×*S*^ → ℝ^*D*^, responsible for mapping initial time windows into embeddings of size *D*. Subsequently, these embeddings processed by an MLP-based model *g*:ℝ^*D*^ → ℝ^*Y*^.

### 3.2 Motivation

**Figure 1:**
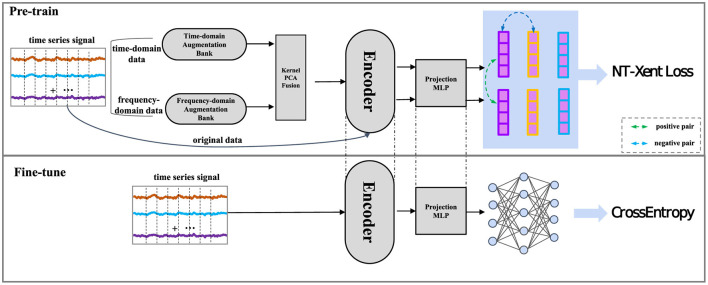
The framework of the Time-Frequency Fusion Contrasting (TF-FC) method consists of three primary components: the augmentation of the initial sample, the encoder network for both augmented and original samples, and the contrastive learning, constrained by the NT-Xent Loss.

Conventional time-domain augmentation techniques, including jitter, scaling, permutation, and masking, effectively capture temporal variations within the data. Nevertheless, these methods are constrained in capturing the frequency-related aspects of time series, which hold significant information. For example, various classes might showcase unique frequency components, like rhythmic arm movements in activities such as walking or running. On the other hand, frequency-domain augmentation techniques like Fourier transformations or wavelet transforms excel at capturing crucial frequency components within the data. Nonetheless, these methods frequently struggle to capture temporal variations present in the data, potentially resulting in information loss and diminished model accuracy. However, frequency-domain augmentation techniques, like Fourier transformations or wavelet transforms, excel in capturing crucial frequency components present in the data. Yet, they often need to catch up on capturing temporal variations, potentially resulting in information loss and reduced model accuracy. Several methodologies incorporated frequency domain characteristics to enrich the learning of time-series representations in the embedding layer, which may result in information loss, potentially undermining the model's effectiveness by reducing its ability to capture essential data patterns. Hence, there exists a necessity to leverage the advantages of time-domain and frequency-domain methodologies to encompass both temporal and spectral characteristics inherent in time series data. The TF-FC method merges time-domain augmentation and frequency-domain augmentation to generate a diverse set of samples, which can capture both the time and frequency domains of the signal. The Kernel Principal Component Analysis (KernelPCA) is often used to fuse data due to its ability to handle non-linear relationships in the data, providing a more comprehensive and detailed representation of the underlying patterns, therefore, we use KernelPCA to fuse the time-domain and frequency-domain data. Utilizing these complementary techniques, TF-FC designs a pre-trained model that encapsulates an expanded scope of information, resulting in more generalizability and strengthen robustness in the ultimate time series model, the framework of the proposed method is presented in Figure 1.

### 3.3 The augmentation of original sample

- **Time-domain augmentation bank**: According to the study conducted by Chen et al. (2020), the integration of diverse data augmentation techniques has been shown to enhance the quality of learned embeddings. In this paper, we introduce an augmentation bank composed of a collection of straightforward time-domain augmentations. With a set of time-domain augmentations *A* = {*a*_1_, *a*_2_, …, *a*_*K*_}, each augmentation is applied to every signal with a probability of occurrence represented by *p*. In this paper, we adopt four commonly used time domain augmentation techniques and four commonly used frequency domain augmentation techniques, therefore, the probability *p* for applying augmentations is set to 0.25. The list of simple augmentations utilized in this study is provided below:

- Jittering: Add random Gaussian noise to original signals to create new, slightly perturbed versions of signals. Jittering helps enhance the robustness of signal processing by simulating real-world noise.
- Scaling: An augmentation that multiplies input signals with values sampled from the normal distribution. Scaling can introduce variability in the amplitude or magnitude of the signals to generate the scaled versions of the original signals.
- Permutation: Splits input signals into a certain number of intervals and randomly permutes them. Permutation aims to introduce temporal disorder or rearrangement of original data.
- Masking: Obscuring segments of the signals with a predefined mask value. Masking helps improve a model's ability to handle missing or incomplete data to make it robust.

- **Frequency-domain augmentation bank**: The initial time series data transforms frequency domain data using the Fast Fourier Transform (FFT). In this paper, we introduce an augmentation bank composed of straightforward frequency-domain augmentations. With a set of frequency-domain augmentations *A* = {*a*_1_, *a*_2_, …, *a*_*K*_}, each augmentation is applied to every frequency signal with a probability of occurrence represented by *p*. The list of simple augmentations utilized in this study is provided below:

- Low-pass filter: A low-pass filter selectively allows signals below a specific frequency threshold to pass while attenuating higher frequencies, commonly employed to reduce noise or emphasize lower-frequency components in signal processing.
- Phase shift: Gaussian noise perturbs the phase spectrum values, while the phase shift augmentation introduces a random value ranging from −π to π to the existing phase values.
- Remove frequency: This method selectively alters the input data by applying a binary mask generated with a specified perturbation ratio, effectively zeroing elements based on this mask, thereby serving as a method to remove components for time series selectively.
- Add frequency: The technique involves the introduction of perturbations to data by utilizing a binary mask, providing a method for controlled alterations within the dataset, thereby serving as a method to selectively add components for time series.

- To obtain the final augmented data, we employ the kernel PCA method to combine the time-domain augmented time series and frequency-domain augmented time series. Firstly, the time-domain augmented time series and frequency-domain augmented time series undergo flattening operations to reshape them into one-dimensional arrays while preserving their channel and sequence length information. Specifically, for the time-domain augmented time series, it typically consists of a two-dimensional array, where one dimension represents the time steps, and the other dimension represents the signal channels. Similarly, for the frequency-domain augmented time series, it also typically comprises a two-dimensional array, with one dimension representing the frequency components and the other dimension also representing the signal channels. During the flattening operation, these two-dimensional arrays are reshaped into one-dimensional arrays by concatenating all time steps or frequency components into a single continuous sequence. Subsequently, KernelPCA transformations are individually applied to both flattened datasets, generating transformed data feature representations separately. The next stage involves concatenating these transformed representations to form an integrated feature space. Finally, the reshaped representation constitutes the final augmented time series, now enriched with combined temporal and frequency domain information obtained through the KernelPCA fusion process. Specifically given two augmented time series ***data***^t_aug^ and ***data***^f_aug^, then the augmented data were flattened to ***data***^t_aug_flat^ and ***data***^f_aug_flat^. The final augmented data is obtained as follows: ***data***^final^ = ***KernelPCA***(***data***^t_aug_flat^)⊕***KernelPCA***(***data***^f_aug_flat^).

### 3.4 Encoder

In our study, we employ a 3-layer ResNet as the foundational structure for our self-supervised learning framework's encoder component. The ResNet architecture's widespread adoption in computer vision tasks stems from its proficiency in managing deep neural networks containing numerous layers while avoiding the challenges posed by the vanishing gradient problem. Specifically in the domain of time series data, 1D ResNet models have proven effective in capturing temporal dependencies and producing meaningful embeddings.

### 3.5 The contrastive learning

Self-supervised learning involves training an encoder without employing explicit target labels. To achieve this goal, pretext tasks are employed, among which contrastive approaches specifically try to align diverse views of identical instances by employing metric learning objective functions. The objective of self-supervised learning is to acquire meaningful and valuable data representations, which subsequently utilize in downstream tasks like classification and prediction.

The contrastive learning method proposed in this paper establishes the final augmented data and the original data as positive pairs while generating negative samples by sampling from different instances within the same batch. The normalized temperature-scaled cross-entropy loss (NT-Xent) serves as the chosen objective function for training the model. Mathematically, let ***z***_*i*_ and ***z***_*j*_ represent the representations of two samples within the batch, where *i* and *j* denote the indices of these samples. The formula for the NT-Xent loss is expressed as Equation (1).


(1) $${ℒ}_{i,j}=-\log\frac{\exp(\mathrm{sim}(z_{i},z_{j})/\tau )}{\sum_{k=1}^{2N}1_{\left[k\neq i\right]}\exp(\mathrm{sim}(z_{i},z_{k})/\tau )}$$

Here, τ denotes the temperature parameter, 1_[*k*≠*i*]_ represents an indicator function that equals 1 when *k*≠*i* and 0 otherwise, and sim(·, ·) denotes the cosine similarity function. The NT-Xent loss promotes the embeddings of positively paired samples to be proximate in the embedding space while urging the embeddings of negatively paired samples to be distant from each other. Throughout the training process, the model is trained to maximize the average NT-Xent loss across all positive and negative pairs within the batch, the loss function is shown in Equation (2).


(2) $$\begin{array}{l} L_{NT}=\frac{1}{N}\overset{N}{\underset{i=1}{\sum }}\frac{1}{2}\left(L_{i,i^{\prime }}+L_{i^{\prime },i}\right) \end{array}$$

where *N* is the batch size, *i*′ is the index of the augmented view of the same instance as *i*. The equation computes the mean NT-Xent loss over all positive and negative pairs contained in the batch. By optimizing the NT-Xent loss, the model acquires the ability to extract meaningful and valuable features from the data, subsequently applicable for downstream tasks like classification and prediction.

## 4 Experiment

### 4.1 Datasets and preprocessing

In order to evaluate the effectiveness of the proposed approach, a comprehensive set of experiments was performed on four distinct publicly available time series datasets from various domains, including SleepEEG (Goldberger et al., 2000), HAR (Micucci et al., 2017), Gesture (Liu et al., 2009), and Epilepsy (Andrzejak et al., 2001), Table 1 illustrates the classification number, division proportion, and window size of the datasets.

**Table 1:** Briefly description and operation of four domains datasets.

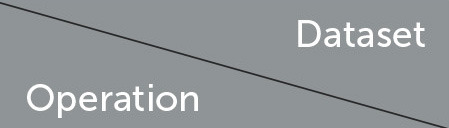	**SleepEEG**	**HAR**	**Gesture**	**Epilepsy**
Number of classification	5	17	8	2
Ratio of pretrain	60%	60%	60%	60%
Ratio of finetune	20%	20%	20%	20%
Ratio of test	20%	20%	20%	20%
Sliding window size	3000	171	206	178
Overlap rates	50%	50%	75%	75%

- **SleepEEG dataset:** The SleepEEG domain dataset we used is PhysioBank, which comes from Goldberger et al. (2000). Sleep-EDF includes whole-night PSG sleep recordings, where we used a single EEG channel (i.e., Fpz-Cz). The dataset contains 153 whole-night sleeping electroencephalography (EEG) recordings produced by a sleep cassette. Every sample is associated with one of the five sleeping patterns/stages: Wake (W), Non-rapid eye movement (N1, N2, and N3), and Rapid Eye Movement (REM). The dataset is sampled at 100 Hz.
- **HAR dataset:** The HAR domain dataset we used is UniMib-SHAR, which researchers at the University of Milano-Bicocca collected. Its primary purpose is to detect various “falling” activities. This dataset comprises information gathered from 30 individuals aged between 18 and 60 years old, utilizing Android smartphones. Throughout the data collection phase, participants were required to carry smartphones in both their left and right pockets. Sensor signals were sampled at a rate of 50 Hz.
- **Gesture dataset:** The dataset contains accelerometer measurements of eight simple gestures that differ based on the paths of hand movement. The eight gestures are hand swiping left, right, up, and down, hand waving in a counterclockwise or clockwise circle, hand waving in a square, and waving a right arrow. The classification labels are these eight different kinds of gestures. The dataset uses three channels corresponding to three coordinate directions of acceleration and is sampled at 100 Hz.
- **Epilepsy dataset:** The dataset contains single-channel EEG measurements from 500 subjects. For every subject, the brain activity was recorded for 23.6 s. The dataset was then divided and shuffled (to mitigate sample-subject association) into 11,500 samples of 1 s each. The raw dataset features five classification labels corresponding to different states of subjects or measurement locations—eyes open, eyes closed, EEG measured in the healthy brain region, EEG measured in the tumor region, and whether the subject has a seizure episode. To emphasize the distinction between positive and negative samples in terms of epilepsy, We merge the first four classes into one, and each time series sample has a binary label describing if the associated subject is experiencing a seizure.

### 4.2 Implementation details

#### 4.2.1 Augmentation

Within the time-domain augmentation module, four techniques are employed: jittering, scaling, permutation, and masking. Specifically, for jittering, Gaussian noise with a standard deviation of 0.01 is added to the time series data. Scaling operates by multiplying data points with a random scalar value from the range (0.9, 1.1). Permutation randomly rearranges the order of the time series data points. Masking obscures 10% of the time series data points. In the frequency-domain augmentation module, we use four techniques: Low-pass filter, phase shift, remove frequency, and add frequency. Low-pass filter Phase shift involves that phase spectrum values are perturbed by Gaussian noise, then adds a random value between -π and π to the phase values; we choose a random value between -π and π for every signal sample. Remove frequency involves 10% of the elements of the frequency data that will be randomly zeroed out. Add frequency involves 10% of the elements of the frequency data that will be randomly perturbed by adding noise.

#### 4.2.2 Pretext setup

The encoder undergoes pre-training within the suggested contrastive learning framework, employing the Adam optimizer (Kingma and Ba, 2015) with a learning rate set at 10-4 and decay rates of 0.9 and 0.99, sustained for 100 epochs. This optimizer can be a good choice for time series classification problems, particularly those with complex and varied patterns in the data. We also add L2 regularization to the loss function to mitigate over-fitting. As different datasets might present varying input data lengths, it's crucial to ensure uniform feature lengths extracted from these inputs. Adaptive average pooling is employed to standardize the vector length of the ResNet features for this purpose. The projection MLP receives the encoder output and performs projection into a lower-dimensional space, achieved through two fully connected layers equipped with batch normalization and ReLU activation functions.

#### 4.2.3 Fine-tuning

The prediction model comprises two hidden layers, the first with 256 neurons and the second with 128 neurons, utilizing ReLU activation functions. Additionally, there's dropout regularization applied with a probability of 0.2. For optimization, the model employs the Adam optimizer with default parameters: ϵ = 10^−4^, β_1_ = 0.9, and β_2_ = 0.99. The output layer uses the softmax activation function.

### 4.3 Evaluation metrics

The evaluation of the model's performance is based on three metrics:

- **Accuracy**: This metric measures the ratio of correctly predicted instances to the total instances in the dataset, providing an overall assessment of the model's correctness.
- **Precision**: Precision quantifies the accuracy of the positive predictions made by the model. It is calculated as the ratio of true positive predictions to the total positive predictions (true positives + false positives).
- **F1-score**: The F1-score is the harmonic mean of precision and recall. It gives a balance between precision and recall, providing a single score that considers both false positives and false negatives.

The calculation formula of accuracy, precision and F1-score are as follows:


$$\text{Accuracy}=\frac{\text{Number of Correct Predictions}}{\text{Total Number of Predictions}}$$

$$\text{Precision}=\frac{\text{True Positives}}{\text{True Positives + False Positives}}$$

$$\text{F1-score}=2\times \frac{\text{Precision }\times \text{ Recall}}{\text{Precision }+\text{ Recall}}$$

### 4.4 Experimental results

In this study, we employed a total of seven baseline methods. To examine the utility of pre-training, we consider two additional approaches applied directly to fine-tuning datasets without any pre-training: Non-DL (a non-deep learning KNN model) and Random Init (randomly initializes the fine-tuning model). Additionally, we utilized five self-supervised state-of-the-art (SOTA) methods, including TF-C (Zhang et al., 2022), TS2vec (Yue et al., 2022), Mixing-up (Wickstrøm et al., 2022), TS-TCC (Eldele et al., 2021), and SimCLR (Tang et al., 2020).

Below are introductions to these baseline methods.

- **TS2Vec**: This method introduces the concept of contextual consistency and employs a hierarchical loss function to capture the long-range structure in time series data.
- **Mixing-up**: This method introduces innovative mix-up augmentation and pretext tasks, designed to accurately predict the mixing proportion of two time series samples.
- **TS-TCC**: The method uses temporal and contextual contrastive learning to help the model learn consistent features across variations and identify distinct feature changes over different time intervals.
- **TF-C**: The method proposes a novel contrastive learning approach called temporal-frequency consistency, where data from the temporal domain and the frequency domain are treated as positive samples.
- **SimCLR**: The method adapts the SimCLR contrastive learning framework for the domain of human activity recognition, utilizing eight specialized data augmentation techniques designed for time series data.

**Figure 2:**
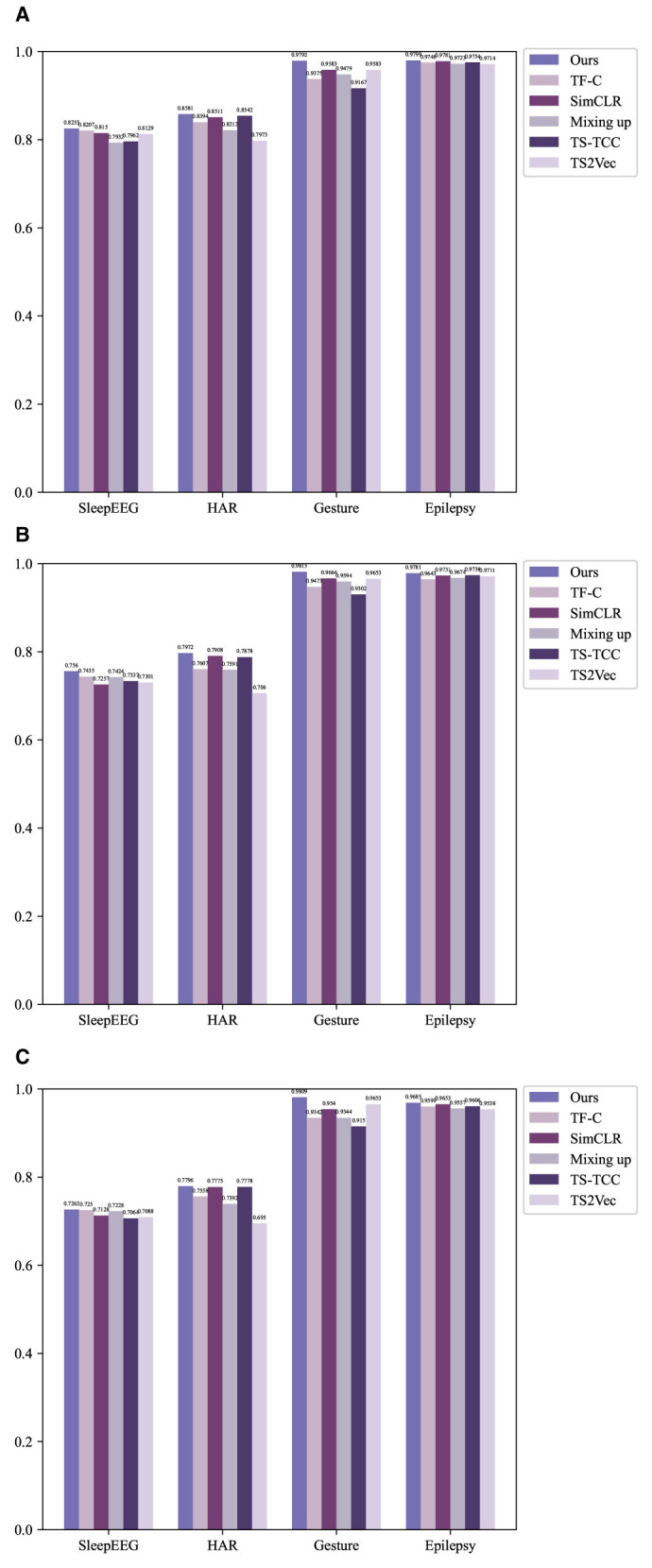
Experimental results compared with other methods using histograms. **(A)** Accuracy. **(B)** Precision. **(C)** F1-score.

The experimental results are shown in the Table 2 and Figure 2.

**Table 2:** Experimental results.

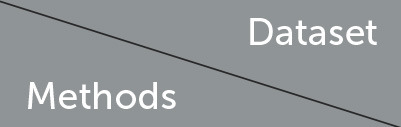	**SleepEEG**			**HAR**			**Gesture**			**Epilepsy**		
	**Evalution metrics**	**Acc**	**Precision**	**F1-score**	**Acc**	**Precision**	**F1-score**	**Acc**	**Precision**	**F1-score**	**Acc**	**Precision**	**F1-score**
Random init.	0.7682	0.6921	0.6769	0.7170	0.5932	0.5543	0.8333	0.8499	0.8181	0.8991	0.9400	0.8027
KNN	0.7509	0.6961	0.6488	0.6905	0.6369	0.6192	0.7917	0.8510	0.7783	0.9326	0.9568	0.8792
TF-C	0.8207	0.7435	0.7250	0.8394	0.7607	0.7558	0.9375	0.9473	0.9342	0.9746	0.9643	0.9599
SimCLR	0.8150	0.7257	0.7128	0.8511	0.7908	0.7775	0.9583	0.9664	0.9540	0.9781	0.9731	0.9653
Mixing up	0.7932	0.7424	0.7228	0.8212	0.7591	0.7392	0.9479	0.9594	0.9344	0.9723	0.9674	0.9557
TS-TCC	0.7962	0.7337	0.7064	0.8542	0.7878	0.7778	0.9167	0.9302	0.9150	0.9754	0.9738	0.9606
TS2Vec	0.8129	0.7301	0.7088	0.7973	0.7060	0.6950	0.9583	0.9653	0.9653	0.9714	0.9711	0.9538
TF-TC (ours)	**0.8253**	**0.7560**	**0.7262**	**0.8581**	**0.7972**	**0.7796**	**0.9792**	**0.9815**	**0.9809**	**0.9799**	**0.9781**	**0.9685**

Bold values signify accuracy, precision, and F1-score.

The table above showcases the experimental results of various methods across diverse domain datasets. In terms of experimental evaluation metrics, we use accuracy, precision, and F1-score for comprehensive evaluation of the model. To assess the efficacy of pre-training, we conducted a comparative analysis involving two additional methodologies, which directly use fine-tuning datasets without any pre-training. The initial approach, denoted as “Non-DL,” employs a non-deep learning K-nearest neighbors (KNN) model. The subsequent method, named “Random init.,” involves the random initialization of the fine-tuning model. TF-FC (ours) showcases promising results, achieving the highest accuracy, precision, and F1-score in all different domain datasets, which displays the robustness of the model. Specifically, in the SleepEEG dataset, TF-TC secures the highest accuracy at 82.53%, coupled with precision and F1-score reaching 75.21 and 72.62%, respectively. Similarly, in the HAR dataset, TF-TC demonstrates substantial prowess with an accuracy of 85.81% and precision and F1-score at 79.72 and 77.96%, respectively. The Gesture dataset further solidifies TF-TC's dominance, achieving an accuracy of 97.92% alongside precision and F1-score metrics of 98.15 and 98.09%, respectively. In the Epilepsy dataset, TF-TC secures the highest accuracy at 97.99%, coupled with precision and F1-score reaching 97.81 and 96.85%, respectively. The superior performance of TF-FC across these four datasets can be attributed to several key factors. Firstly, TF-FC leverages time-frequency fusion techniques, enabling it to effectively integrate temporal and spectral information. Secondly, TF-FC employs comprehensive data augmentation strategies, enhancing the diversity of training samples and facilitating robust model training. Lastly, TF-FC incorporates kernel PCA fusion for nonlinear feature extraction, allowing it to capture complex patterns inherent in the data.

Overall, the analysis indicates that TF-TC outperforms other methods in all metrics across SleepEEG, HAR, Gesture, and Epilepsy datasets, showcasing its efficacy in different tasks of time series classification.

### 4.5 Ablation study

The ablation experiment in this study aims to investigate the contributions of time-domain augmentation and frequency-domain augmentation to the performance of the TF-FC method. For this purpose, two experiments were carried out: one excludes time-domain augmentation and solely relies on frequency-domain techniques, while the other involved omitting frequency-domain augmentation and exclusively employing time-domain techniques. The experimental results are shown in the Table 3.

**Table 3:** Ablation experiment results.

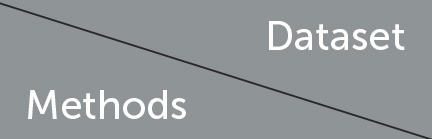	**SleepEEG**			**HAR**			**Gesture**			**Epilepsy**		
	**Evaluation metrics**	**Acc**	**Precision**	**F1-score**	**Acc**	**Precision**	**F1-score**	**Acc**	**Precision**	**F1-score**	**Acc**	**Precision**	**F1-score**
Only time-domain	0.8070	0.7448	0.7194	0.8016	0.7395	0.6982	0.9583	0.9664	0.9540	0.9705	0.9742	0.9519
Only freq-domain	0.8201	0.7539	0.7047	0.7852	0.7090	0.6814	0.9271	0.9399	0.9241	0.9411	0.9592	0.8969
TF-TC (ours)	**0.8253**	**0.7560**	**0.7262**	**0.8581**	**0.7972**	**0.7796**	**0.9792**	**0.9815**	**0.9809**	**0.9799**	**0.9781**	**0.9685**

Bold values signify accuracy, precision, and F1-score.

The results shown in Table 3 illustrate the ablation experiments conducted by independently evaluating the time-domain augmentation and frequency-domain augmentation modules. The TF-FC method, leveraging both time-domain and frequency-domain augmentations, demonstrates superior performance, yielding the highest accuracy, precision, and F1 scores across all four datasets. Notably, removing either augmentation module leads to a decline in accuracy, precision, and F1 scores, underscoring the substantial contributions of both modules to the TF-FC method's performance. Indeed, the varying performances of the “only Time-domain” and “only Frequency-domain” approaches across datasets highlight the nuanced importance of time-related and frequency-domain information in different domains. The proposed TF-TC method offers a unique solution that combines the strengths of both time and frequency domains. By integrating Time-Frequency Fusion Contrasting, TF-TC effectively leverages the complementary nature of these domains, allowing for a more comprehensive representation of the underlying data characteristics. These observations emphasize the method's adept utilization of the complementarity between time-domain and frequency-domain augmentations, culminating in enhanced self-supervised learning for time series classification.

### 4.6 Visualizations

**Figure 3:**
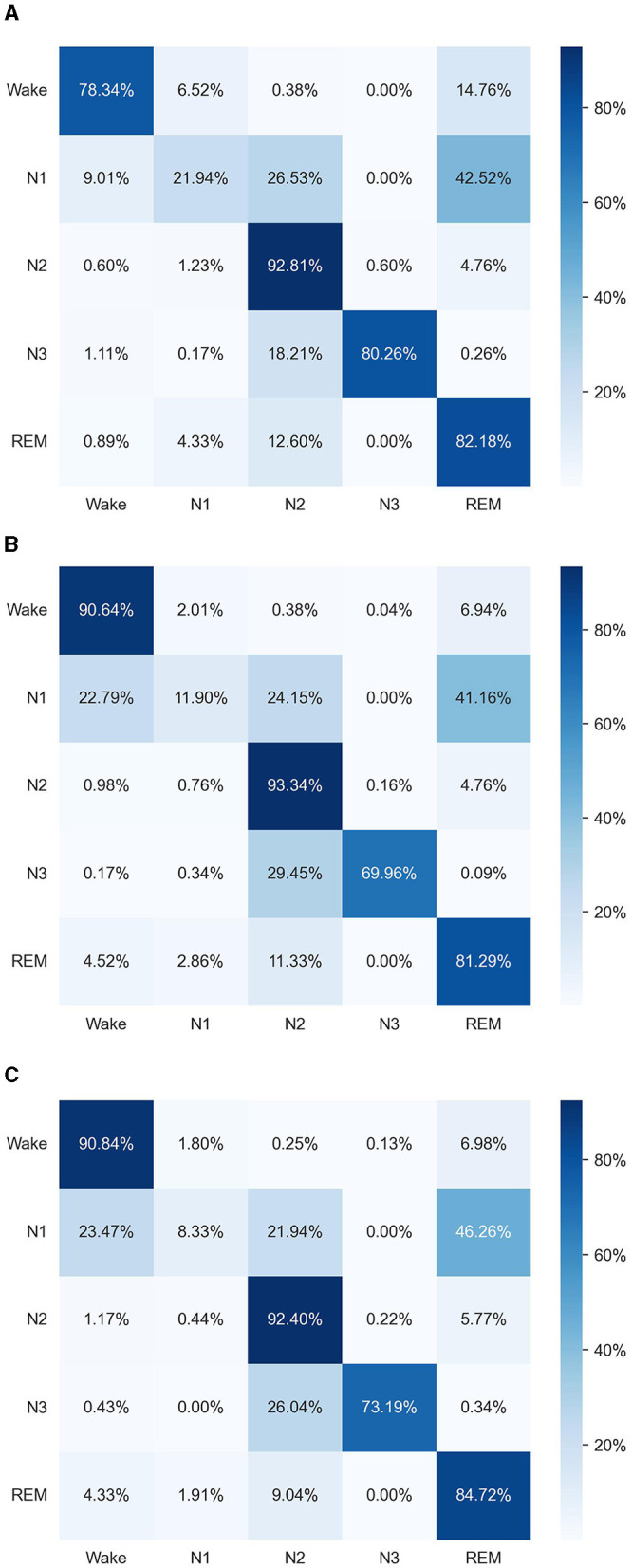
Experimental results compared with other methods using histograms. **(A)** Only time-domain augmentation. **(B)** Only frequency-domain augmentation. **(C)** TF-FC (ours).

For visualizing the performance of our method, we generated three confusion matrices specific to the SleepEEG dataset, as shown in Figure 3. The first confusion matrix shows the results of only using the time-domain augmentation module, the second only using the frequency-domain augmentation module, and the third using the TF-FC (ours) method.

The overall performance of combining the time-domain augmentation module and frequency-domain augmentation module is better than only using the time-domain augmentation module or the frequency-domain augmentation module. Specifically, our method showcases superior performance compared to using only time-domain augmentation, especially within the “Wake” class; however, slight decreases are observed in certain classes like “N1” and “N3,” which could be due to the introduction of noise or irrelevant features during the frequency-domain augmentation process. Additionally, the complex interplay between time-domain and frequency-domain features may result in trade-offs in performance across different classes. Moreover, when directly compared to using only time-domain augmentation, our method outperforms three out of five categories, although displaying slightly lower performance in “N1” and “N2.” Our method outperforms only the use of time-domain augmentation three out of five but is slightly worse in “N1” and “N2.” “N1” and “N2” stages often exhibit complex patterns and transitions between sleep states, which may pose challenges for the model to accurately distinguish between them. The addition of frequency-domain augmentation may not necessarily enhance the model's ability to capture these subtle variations, leading to comparable or slightly lower performance compared to using only time-domain features.

The combination of both time-domain and frequency-domain augmentations within our method showcases superior performance compared to individual approaches, showing its comprehensive advantage in overall performance.

## 5 Conclusion

This paper introduces an innovative approach called the Time-Frequency Fusion Contrasting (TF-FC) method, designed specifically for self-supervised time series classification. TF-FC utilizes the potency of contrastive learning to address the common problem of demanding extensive labeled data. Combining time-domain and frequency-domain augmentations generates a diverse array of samples, effectively capturing both the temporal nuances and spectral attributes inherent in time series data. The results obtained from experiments on four benchmark datasets strongly support the effectiveness of the TF-FC method. It showcases state-of-the-art performance, surpassing conventional machine learning techniques and other self-supervised approaches. These outcomes validate the TF-FC method's potential and emphasize the benefits derived from integrating both time-domain and frequency-domain augmentations, significantly boosting the model's capabilities. But the computational complexity of TF-FC may pose challenges due to the intensive processing power and memory resources required for combining time-domain and frequency-domain augmentations. The TF-FC method demonstrates substantial promise for real-world applications in health assessment, accident monitoring, and various other domains. For instance, in healthcare, TF-FC could be utilized for analyzing physiological signals such as EEG or ECG data, aiding in the diagnosis of neurological disorders or monitoring patients' health status over time. Similarly, in accident monitoring systems, TF-FC could contribute to the early detection and prediction of critical events based on sensor data, thereby enhancing safety measures and preventing potential accidents. In the absence of extensive labeled data, the method demonstrates favorable performance. Subsequent research avenues could delve into integrating supplementary data sources or modalities, like contextual environmental factors, aiming to advance the method's performance and applicability. Moreover, extending the TF-FC approach to address diverse time-series classification tasks could facilitate training a versatile model using large amounts of unlabeled datasets. This could further transfer knowledge to smaller datasets with limited or zero labels, enhancing the model's adaptability across domains.

## Data availability statement

The original contributions presented in the study are included in the article/Supplementary material, further inquiries can be directed to the corresponding authors.

## Author contributions

WZ: Formal analysis, Project administration, Writing - original draft. LF: Conceptualization, Supervision, Writing - review & editing.
